# WRNing for the right DNA repair pathway choice

**DOI:** 10.18632/aging.204120

**Published:** 2022-06-01

**Authors:** Jong-Hyuk Lee, Deborah L. Croteau, Vilhelm A. Bohr

**Affiliations:** 1 Department of Biomedical Sciences, Mercer University School of Medicine, Savannah, GA 31404, USA; 2 DNA Repair Section, National Institute on Aging, National Institutes of Health, Baltimore, MD 21224, USA

**Keywords:** DNA repair, RecQ helicase, helicase, DNA double strand repair pathways

Aging is an inevitable fate of all living organism. Nine characteristics, also known as hallmarks of aging, have been identified to better understand the true nature of the aging process, and to possibly prevent or even revert it [1]. Genomic instability is one of these traits, leading to genetic mutations and increased risk of cancer during cellular/organismal aging.

Premature aging diseases, also called ‘progeroid syndrome’, display signs and features of normal aging in early life, ultimately leading to premature death. Although progeroid syndromes do not perfectly mimic chronological aging they can be excellent model systems to study characteristics of normal aging. Werner syndrome (WS) is one of the rare autosomal recessive progeroid syndromes, characterized by accelerated in vivo/in vitro aging [2]. WRN is suggested to play a central role in maintaining genome stability and rapidly recruits to the DNA damage sites to take part in DNA repair, including base excision DNA repair (BER), classical/alternative non-homologous end joining (NHEJ), homologous recombination (HR), and replication re-start after DNA damage.

WRN makes critical DNA-repair pathway choices between classical and alternative NHEJs [3]. In addition to its key role in NHEJ, WRN has been suggested to also participate in HR. However, it was still unclear how it regulates the pathway choice between NHEJ and HR.

In a recent paper, we showed that CDK2 phosphorylating WRN on serine residue 426 is critical for WRN to make its DNA double strand break (DSB) repair pathway choice between NHEJ and HR [4]. Abnormal DSB recruitment, altered interaction with RPA, strand annealing activity, and DSB repair activities were observed when cells were forced to express WRN engineered to mimic the unphosphorylated or phosphorylation state at serine 426. It is likely that the CDK2 phosphorylation on serine 426 stabilizes WRN’s affinity with RPA to increase its long-range resection at the end of DNA strands, a key process for HR initiation. Collectively, the data demonstrated that a CDK2-dependent phosphorylation of WRN regulates DSB repair pathway choice between NHEJ and HR, and that phosphorylation of WRN by CDK2 increases its binding affinity to RPA thereby possibly stabilizing resected single-stranded DNA. These findings along with the previous discovered role in NHEJ pathway choice, move our understandings one step closer to the true nature of genomic instability that lies within WS.

**Figure 1:**
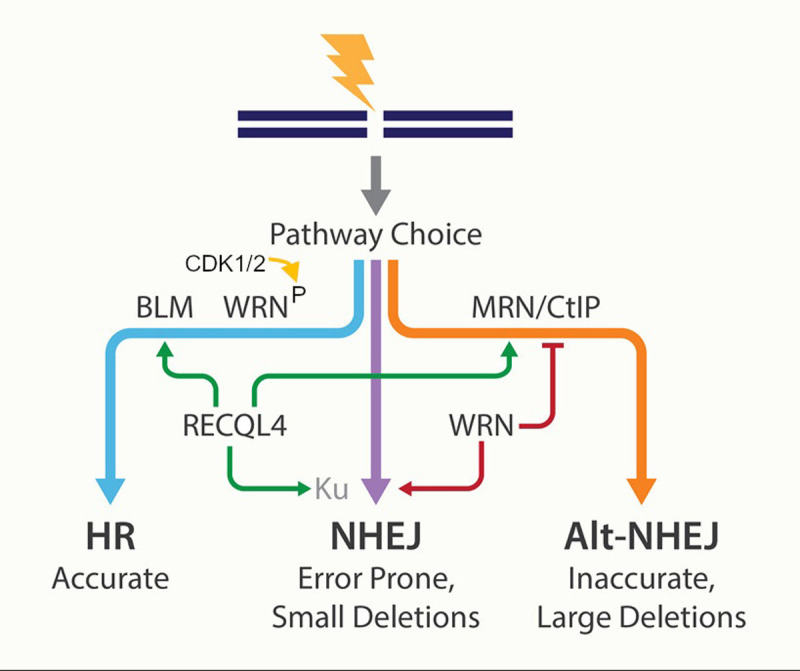
**Points of control by human RecQ helicases in DSB repair pathway choice.** HR, homologous recombination; NHEJ, non-homologous end joining; Alt-NHEJ, alternative non-homologous end joining.

Interestingly, another RECQL family member, RECQL4 has also been identified as one of the crucial decision makers during DSB repair pathway choice [5]. During the S/G2 phase, CDK1 and 2 phosphorylation induce DSB recruitment of RECQL4 by enhancing the binding affinity to MRE11. RECQL4 phosphorylation promoted helicase activity, DNA end resection, and cell survival after ionizing radiation, preventing cellular senescence. A graphic representation of RecQs in DSB pathway choice is shown in Figure 1.

Notably, primary fibroblast cells show similarly increased persistent DNA damage after WRN or RECQL4 knockdown, and CDK regulatory mechanisms on WRN and RECQL4 have functional similarities in DSB response [5]. It is thus conceivable that investigating the cooperative roles of WRN and RECQL4 in DSB pathway choice should be a future goal for DNA repair and aging research.
